# Combined transcriptome and metabolome analyses reveal the effects of selenium on the growth and quality of *Lilium lancifolium*

**DOI:** 10.3389/fpls.2024.1399152

**Published:** 2024-05-17

**Authors:** Xiaogang Jiang, Wuxian Zhou, Darong Li, Hua Wang, Yuying Yang, Jinwen You, Haihua Liu, Lunqiang Ai, Meide Zhang

**Affiliations:** Key Laboratory of Biology and Cultivation of Herb Medicine, Ministry of Agricultural and Rural Affairs, Institute of Chinese Herbal Medicines, Hubei Academy of Agricultural Sciences, Enshi, China

**Keywords:** *Lilium lancifolium*, selenium, soluble sugar, amino acid, soluble protein

## Abstract

*Lilium lancifolium* Thunb (*L. lancifolium*) is an important medicinal and edible plant with outstanding functionality for selenium (Se) biofortification. However, the molecular response of *L. lancifolium* to exogenous Se has not been fully elucidated. In this study, the effects of different levels of Se on *L. lancifolium* growth and quality were explored by transcriptome, metabolome and biochemical analyses. The results showed that the total Se and organic Se content in *L. lancifolium* bulbs increased with increasing Se dosage (0–8.0 mmol/L). Moreover, Se stimulated the growth of *L. lancifolium* at low level (2.0 mmol/L) but showed an inhibitory effect at high levels (≥4.0 mmol/L). Metabolomic and biochemical analyses revealed that the bulb weight and the content of amino acid, soluble sugar, and soluble protein were significantly increased in the 2.0 mmol/L Se treatment compared with those in the control (0 mmol/L Se). Transcriptome and metabolome analyses revealed that the significant upregulation of the *GPD1*, *GPAT* and *ADPRM* genes promoted glycerophospholipid accumulation. Additionally, the significantly upregulated *glyA* and downregulated *asnB*, *nadB*, *thrA* and *SAT* genes coordinate to the regulation of amino acid biosynthesis. The significantly upregulated *SUS, bgl B, BAM*, and *SGA1* genes were involved in soluble sugar accumulation under Se treatment. In summary, this study identified the optimal Se concentration (2.0 mmol/L), which significantly improved the growth and nutritional quality of *L. lancifolium* and contributed to understanding the combined effects of Se treatment on the expression of genes and the accumulation of metabolites in *L. lancifolium* bulbs.

## 1 Introduction

*Lilium lancifolium* Thunb is a perennial herbaceous species belonging to the Liliaceae family and is native to China, Japan and North Korea (Munafo and Gianfagna, 2015; Zhao et al., 2016).

The bulbs of *L. lancifolium* can be used as both medicine and food (Gao et al., 2015). The main chemical components of the edible bulb of *L. lancifolium* include sucrose, polysaccharides, amino acids, vitamins, and minerals (Munafo and Gianfagna, 2015; Wang et al., 2019). As an essential trace element, Se (Se) plays a beneficial role in maintaining the physical health of humans and animals (Wang et al., 2021). Daily intake of appropriate amounts of Se can prevent various diseases, such as Keshan disease, cardiovascular and cerebrovascular diseases, joint inflammation and cancer (Pyrzynska and Sentkowska, 2021).

Generally, appropriate Se is beneficial for plant growth, while excessive Se can inhibit plant growth and produce toxic effects (El-Ramady et al., 2016). A study revealed that a moderate concentration of Se stimulated the growth and antioxidant capacity of *Salvia miltiorrhiza* (Zhang et al., 2023). However, high-dose Se reduced the survival rate and yield of *Atractylodes macrocephala* (Zhou et al., 2021). Moderate Se concentrations could increase the content of nutrients, such as most amino acids and trace elements, thereby improving the quality and economic benefits of plants (Zhu et al., 2017). For instance, Se supplementation can increase the level of exogenous amino acids, including lysine and leucine, in soybean (Zhang et al., 2020). Soluble sugars such as sucrose, fructose, and glucose can be generated through sucrose and starch metabolism (Malik et al., 2011). A study on tomato revealed that Se application could increase the soluble sugar content, thus improving tomato fruit quality (Hu et al., 2023). In addition, foliar Se application can also affect the accumulation of Se and secondary metabolites in plants (da Silva et al., 2020). A study on cabbage revealed that a moderate concentration of Se (0.4 mmol/L) significantly increased the flavonoid content and phenolic acid biosynthesis (Yang et al., 2022). It has been reported that glucosinolate biosynthesis in broccoli is enhanced by a moderate concentration of Se (0.4 mmol/L) (Tian et al., 2016; Rao et al., 2021). However, the influence of Se on the growth and quality of *L. lancifolium* has not been studied yet.

Although many studies have evaluated the effects of Se application on the growth and development of a wide range of plant species, little information is available on the molecular mechanism underlying these biological processes. Combined transcriptome and metabolome technology has improved the understanding of the interactions between genes and nutritional metabolites in food generating plants. For instance, the mechanism through which Se regulates the catabolism and biosynthesis of amino acids and sugars, ultimately promoting the growth of apple plants, was revealed by integrated analysis of the transcriptome and metabolome (Liu et al., 2024). These technologies help researchers understand the regulatory effects of key genes on several important nutrients in foods. However, the changes in the transcriptome and metabolome of Se-treated *L. lancifolium* plants have not been determined. Hence, it is necessary to elucidate the regulatory network between genes and key metabolites through the combination of transcriptome and metabolome analyses in *L. lancifolium* under Se application.

In this study, we treated *L. lancifolium* plants with different concentrations of Se and measured their morphological, physiological and quality traits, including soluble protein, amino acid, soluble sugar, total flavonoid and alkaloid content. An Illumina NovaSeq 6000 and HPLC−MS/MS system were used to perform transcriptome and metabolome analyses of *L. lancifolium* bulbs in the control and Se treatment groups to further construct a network of genes and individual glycerophospholipids, amino acids and soluble sugars. This study will provide novel insights into the effects of Se on the accumulation of crucial components in *L. lancifolium* bulbs and lay a foundation for unraveling the molecular mechanisms of Se in regulating the growth and quality of *L. lancifolium*.

## 2 Materials and methods

### 2.1 Plant materials and treatments

The healthy and uniform *L. lancifolium* plants used in this study were two years old and were planted in plastic pots (20.0 cm diameter, 14.0 cm depth) with appropriate culture soil in a greenhouse. After transplanting for 30 days, 250 mL of different sodium selenite solutions (with Se concentrations of 0, 0.5, 1.0, 2.0, 4.0, or 8.0 mmol/L) were added to each pot for each treatment. After transplanting for 45 days, the same Se dosage was applied again. After 90 days of transplanting, the plants were collected, and the levels of chlorophyll, soluble sugar, soluble protein, Se, total flavonoids and alkaloids were measured. Bulb samples from the control (0 mmol/L) and treatment (2.0 mmol/L) groups (three replicates for each) were collected and processed to transcriptomic and metabolomic analysis.

### 2.2 Determination of the chlorophyll, soluble protein and soluble sugar content

The levels of chlorophyll, soluble protein and soluble sugar in *L. lancifolium* were determined using previous methods (He et al., 2019). For chlorophyll content detection, 0.1 g of leaf tissue was immersed in 95% ethanol and subsequently placed in the dark for 48 h. A UV2600 spectrophotometer was subsequently used to measure the absorbance of the chlorophyll extract. For soluble protein extraction, 0.3 g bulb samples were homogenized with 5 mL of 50 mmol buffer solution (containing 0.7% NaH2PO4·2H2O and 1.64% Na2HPO4·12H2O, pH 7.8) and subjected to grinding with a precooled mortar and pestle. Then, the samples were centrifuged at 5000 *×* g for 25 min at 4°C, and the supernatant was collected for determination of soluble protein content via the G-250 Coomassie Brilliant Blue method. The soluble sugar content of the samples was extracted in 80% ethanol in a boiling water bath and determined by anthrone colorimetry with a UV–visible spectrometer at a wavelength of 620 nm.

### 2.3 Total flavonoid and alkaloid content

The flavonoid and alkaloid content was measured and analyzed according to previous methods (Zhang et al., 2021). For the flavonoid content, 0.2 g of bulb powder was collected and sonicated with 10 mL of 50% ethanol for 30 minutes. Then, the samples were centrifuged at 10000 rpm for 25 minutes, and 5 mL of the supernatant was collected in a 100 mL beaker supplemented with 8 mL of 1.5% AlCl3 solution and 4 mL of 0.1 mol/L acetic acid sodium acetate buffer (pH 5.5). Immediately, 50% ethanol aqueous solution was added to the mixture, after which the mixture was added to a 25 mL volumetric flask. After 30 minutes, the concentration of the mixed solution was determined via spectrophotometry. For alkaloid analysis, 0.2 g of bulb powder was collected. Then, 1 mL of ammonia water and 10 mL of methanol were added to the mixture, and the mixture was sonicated for 30 minutes twice. The supernatant was filtered using qualitative filter paper and placed in a 90 mm diameter evaporating dish. The filtrate was then evaporated in an 85 °C water bath. Then, 10 mL of chloroform was added to the evaporating dish containing methanol. The mixture was transferred to the colorimetric solution and mixed well for 1 minute. Finally, the mixture was transferred to a separating funnel, and the chloroform layer was measured using a UV-1800 ultraviolet spectrophotometer (Shimadzu, Japan).

### 2.4 Total Se and organic Se content

The total Se concentration in the bulbs of *L. lancifolium* was determined by the HNO_3_-HClO_4_ digestion method described by Deng et al. (2017).

### 2.5 Transcriptomic sequencing and analysis

Total RNA was extracted from the bulb samples using TRIzol reagent (Invitrogen, Carlsbad, CA, USA). Then, RNA quality was determined by a 5300 Bioanalyzer (Agilent) and quantified using an ND-2000 (NanoDrop Technology). Approximately 1 µg of total RNA was used to construct a transcriptome library using the NEBNext® Ultra™ RNA Library Prep Kit. Double-stranded cDNA was generated using a double-stranded cDNA synthesis kit. The libraries were sequenced using a NovaSeq 6000 sequencer. The transcripts were annotated by the Kyoto Encyclopedia of Genes and Genomes (KEGG), Gene Ontology (GO), NCBI protein nonredundant (NR), and Clusters of Orthologous Groups of proteins (COG) databases. Significantly DEGs were defined as those with a |log2FC|≥1 and an FDR ≤ 0.05. In addition, GO and KEGG functional analyses were used to screen significantly enriched GO terms and metabolic pathways. GO and KEGG enrichment analyses were carried out by Goatools and KOBAS, respectively. All the pathway enrichment analysis were performed based on the KEGG database.

### 2.6 Metabolite detection and analysis

Four hundred microliters of extraction solution [methanol: water = 4:1 (v:v)] was used for metabolite extraction. The bulb samples were dried and ground into powder, followed by ultrasonic extraction for 30 min. The samples were subsequently stored at -20°C for 30 min and centrifuged for 15 min (4°C, 13000 × g), after which the supernatant was used for LC–MS/MS analysis. LC−MS/MS analysis of the samples was performed on a Thermo UHPLC-Q Exactive HF-X system equipped with an ACQUITYHSS T3 column. Differentially abundant metabolites with a fold change (FC) ≥ 2 or ≤ 0.5 and a VIP ≥ 1 were identified.

### 2.7 Real-time quantitative PCR analysis

RT−qPCR was carried out to validate the transcriptomic data according to previous methods (Jiang et al., 2023). The *glyceraldehyde-3-phosphate dehydrogenase* (*GAPDH*) gene was used as the reference gene. The 2^−DDCT^ method was used to calculate the relative expression levels of the genes (Livak and Schmittgen, 2001). One-way ANOVA was used to analyze the significant differences between the control and treatment groups.

### 2.8 Statistical analysis

All the data are expressed as the mean values of three biological triplicates ± standard errors. One-way ANOVA was performed to compare the control and Se-treated groups using SPSS 20.0 (SPSS, Inc., Chicago, USA).

## 3 Results

### 3.1 Effects of Se on the growth of *L. lancifolum*

**Figure 1:**
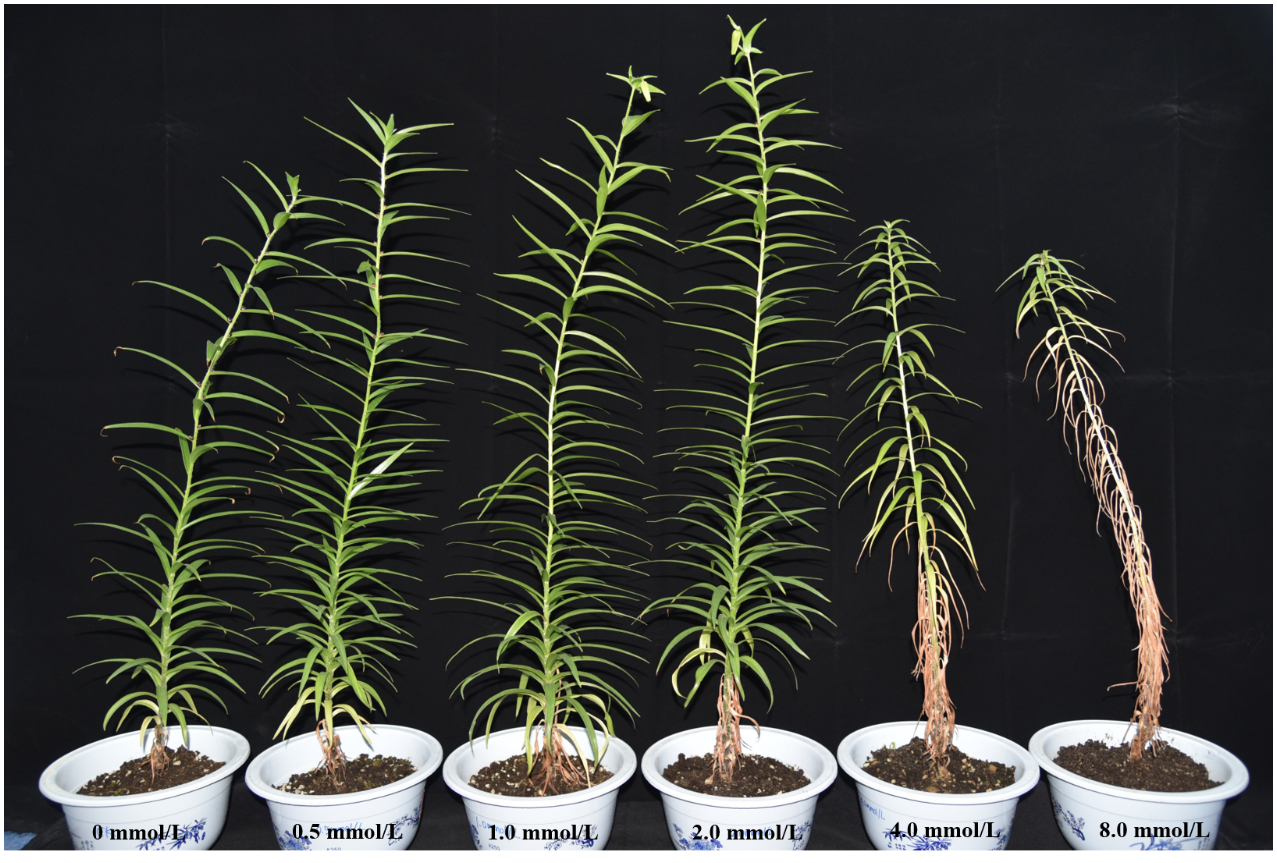
Phenotype of *L. lancifolium* under different Se treatments.

**Figure 2:**
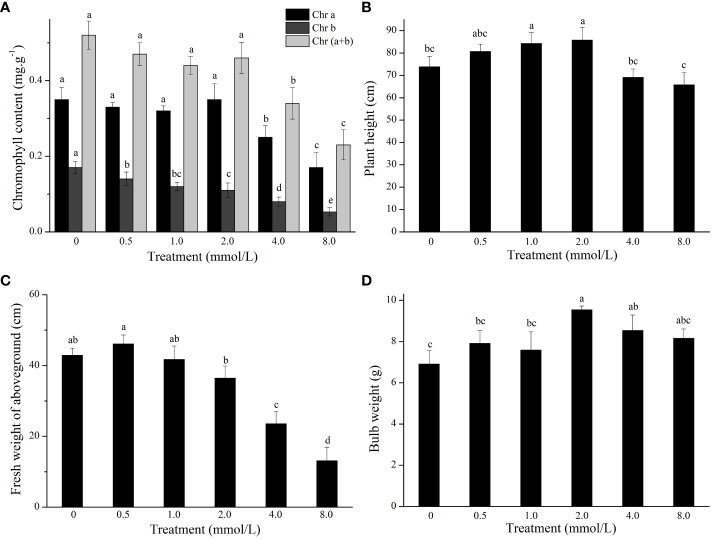
The chlorophyll content **(A)**, plant height **(B)**, fresh weight of aboveground **(C)**, and bulb weight **(D)** of *L. lancifolium* under different Se treatments. Letters mean significant difference at *p < 0.05.*.

The growth of *L. lancifolium* was significantly influenced by Se. As shown in **Figure 1**, 2.0 mmol/L Se effectively promoted the growth of *L. lancifolium*. The plant height and bulb weight were markedly greater than those of the control (**Figures 1**, **2B, D**). More importantly, compared with that of the control (0 mmol/L), the bulb weight of the treatment (2.0 mmol/L) increased by 38.1%. The plant height and bulb weight of the treatments (0.5 and 1.0 mmol/L) were insignificantly greater than those of the control. No significant difference was found in the chlorophyll content or fresh weight aboveground of *L. lancifolium* plants between the 2.0 mmol/L Se treatment group and the control group (**Figures 2A, C**). Treatment with 4.0 mmol/L and 8.0 mmol/L Se strongly inhibited the growth of *L. lancifolium*, and the plants wilted and senesced (**Figure 1**). These results showed that an appropriate concentration (2.0 mmol/L) of Se could facilitate the growth of *L. lancifolium*, but 4.0 mmol/L above Se inhibited the plant growth and even caused the plant death.

### 3.2 Effects of Se on the nutritional quality of *L. lancifolum*

**Figure 3:**
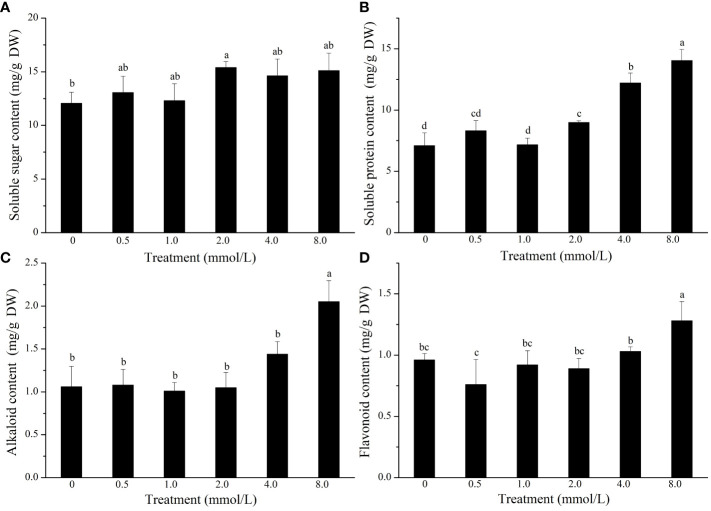
The content of soluble sugars **(A)**, soluble proteins **(B)**, alkaloids **(C)**, and flavonoids **(D)** in *L. lancifolium* bulbs under different Se treatments. Letters mean significant difference at *p < 0.05.*.

To study the effect of Se treatment on the nutritional quality of *L. lancifolium*, the quality traits of the bulbs were investigated. The results indicated that the soluble sugar content was significantly greater in the control treatment than in the 2.0 mmol/L Se treatment. In addition, the soluble protein content in the 2.0–8.0 mmol/L Se treatment group was significantly greater than that in the control group (**Figures 3A, B**). In particular, in the 2.0 mmol/L Se treatment group, the soluble protein content increased by 72.0% compared to that in the control group (**Figure 3B**). Additionally, the flavonoid and alkaloid content did not significantly differ under the 0–4.0 mmol/L Se treatments (**Figures 3C, D**). Moreover, compared with those in the control group, the flavonoid and alkaloid content in the 8.0 mmol/L Se treatment group increased by 33.3% and 93.4%, respectively (**Figures 3C, D**). Generally, the 2.0 mmol/L Se treatment significantly improved the nutritional quality of *L. lancifolium* bulbs.

### 3.3 Effects of Se on the total Se and organic Se content in *L. lancifolum*

*L. lancifolium* can transform Se into considerable amounts of organic Se. As shown in **Supplementary Figure S1**, inorganic selenium was not be detected, and the total inorganic Se was totally transformed into organic Se. The Se content of *L. lancifolium* significantly increased with increasing Na_2_SeO_3_ concentration (0–8.0 mmol/L). The organic Se content in the 0 mmol/L Na_2_SeO_3_ treatment group was 0.9 mg/kg DW, whereas *L. lancifolium* accumulated 2.0 mg/kg DW Se in the 0.5 mmol/L Na_2_SeO_3_ treatment group. The findings showed that Se was taken up by the bulbs of *L. lancifolium* and was extensively transferred to organic Se. High Na_2_SeO_3_ concentrations resulted in high organic Se content in the bulbs. The organic Se concentrations were 3.3, 3.9, 4.7, and 12.5 mg/kg DW in the 1.0, 2.0, 4.0, and 8.0 mmol/L groups, respectively. These results showed that *L. lancifolium* could transform and accumulate a considerable amount of organic Se.

### 3.4 Functional annotations of *L. lancifolum* unigenes

Six cDNA libraries were sequenced from the 0 and 2.0 mmol/L Se treatments. A total of 44,363,384, 45,103,380, 45,909,630, 43,965,638, 56,813,072, and 43,375,422 clean reads were acquired from the CK and T groups, respectively. The average numbers of clean reads obtained from CK and T were 48,051,377 and 45,125,465, respectively. Additionally, the average GC content were 47.87% for CKs and 48.79% for T. The mean Q20 values were 98.37% and 98.09%, and the Q30 values were 95.28% and 94.66% for CK and T, respectively, suggesting the high quality of the transcriptome sequencing data (**Supplementary Table S1**). The raw data were uploaded to the Genome Sequence Archive (GSA) database (CRA014864). Moreover, *de novo* assembly was performed, and 91,435 transcripts and 58,023 unigenes with average lengths of 767 bp and 799 bp, respectively, were identified. All the unigenes are shown in **Supplementary Table S2**.

### 3.5 DEGs in *L. lancifolum* in response to Se treatment

**Figure 4:**
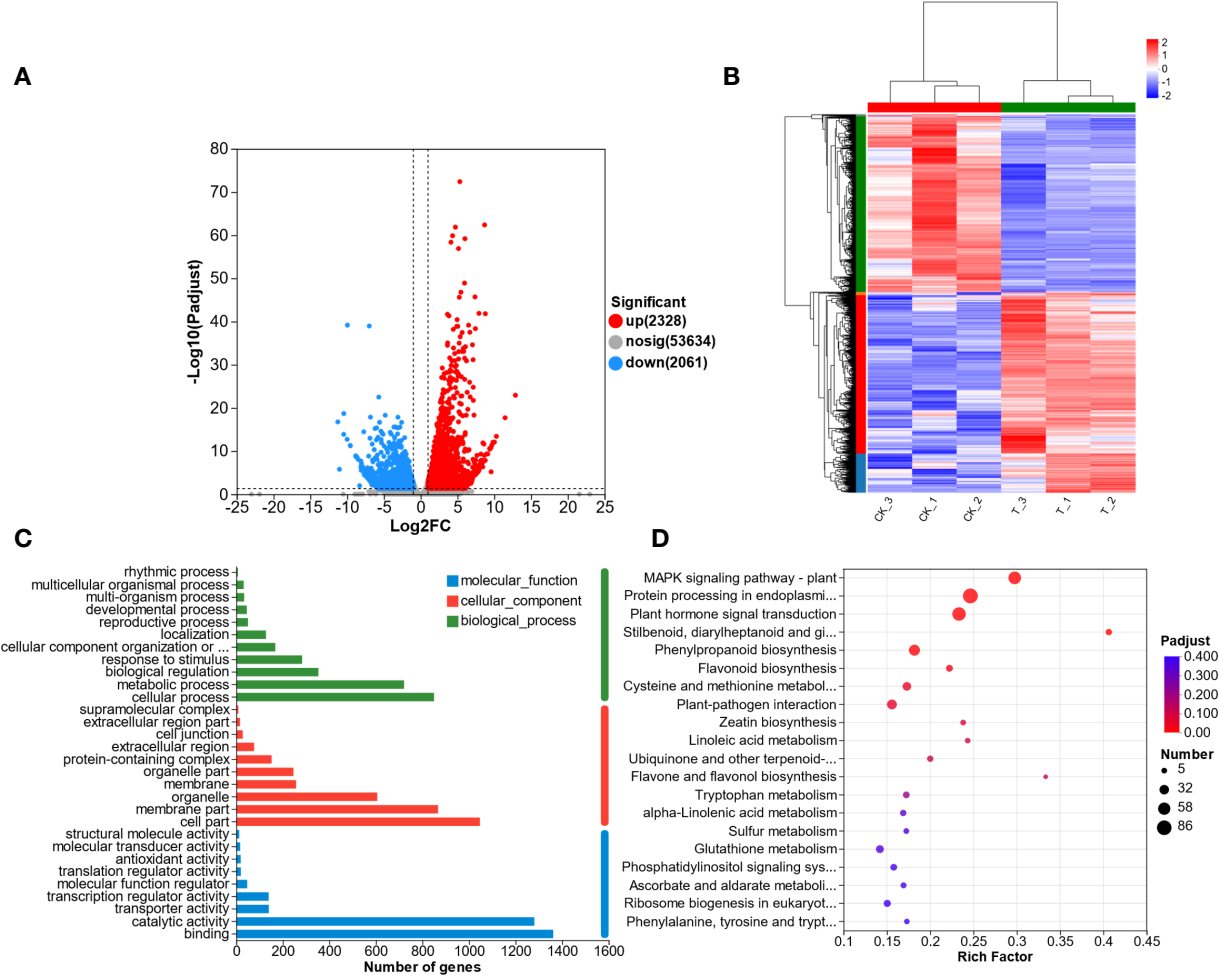
Transcriptome analysis of *L. lancifolium* under 0 and 2.0 mmol/L Se treatments. **(A)** Statistics of the DEGs. **(B)** Hierarchical clustering analysis of DEGs. **(C)** Statistical analysis of the enriched GO terms. **(D)** KEGG pathway enrichment of the DEGs.

To understand the transcriptomic response of *L. lancifolium* to Se treatment, we analyzed the DEGs that were identified in the CK and T groups. A total of 4389 DEGs were screened, including 2328 upregulated DEGs and 2061 downregulated DEGs (**Figure 4A**). In addition, the DEGs from the CK and T groups were clustered using the heatmap clustering method (**Figure 4B**). The DEGs were categorized into two groups, and the expression profiles of the CK and Se-treated plants were different. Genes with similar expression patterns were clustered together. No significant differences were found in the expression patterns of the DEGs between replicates. Furthermore, the DEGs were subjected to GO functional analysis. The DEGs were categorized into molecular function (MF), biological Process (BP), and cellular Component (CC) terms. The significantly enriched MF GO terms were “binding” and “catalytic activity”. In the BP category, “cellular process” and “metabolic process” were significantly enriched. The CC category included DEGs related to cell parts, membranes and organelles (**Figure 4C**; **Supplementary Table S3**). Furthermore, to screen the metabolic pathways enriched in *L. lancifolium* in response to Se, the DEGs were annotated to significantly enriched KEGG pathways. Twenty enriched metabolic pathways were found. The DEGs were significantly.

enriched in cysteine and methionine metabolism (27 DEGs); protein processing in the endoplasmic reticulum (86 DEGs); MAPK signaling pathway-plant (61 DEGs); “stilbenoid, diarylheptanoid and gingerol biosynthesis” (13 DEGs); and flavonoid biosynthesis (16 DEGs) (**Figure 4D**; **Supplementary Table S4**). These results suggested that the DEGs involved in the above metabolic pathways in *L. lancifolium* might be related to soluble protein and amino aid accumuation in *L. lancifolium*.

### 3.6 Metabolomic analysis of *L. lancifolum* treated with Se

**Figure 5:**
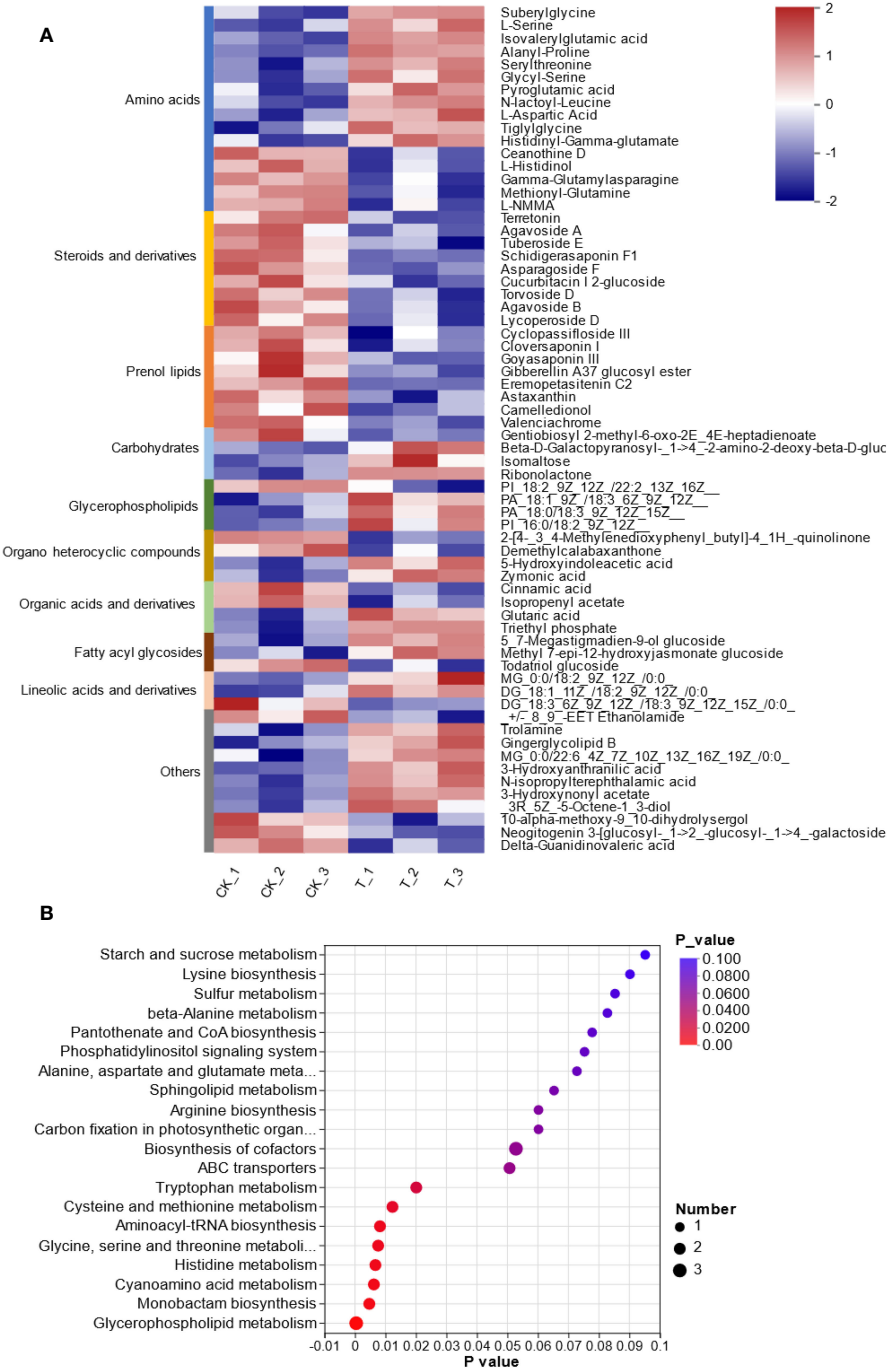
Metabolomic analysis of *L. lancifolium* under 0 and 2.0 mmol/L Se treatments. **(A)** Clustering analysis of the DEMs. **(B)** KEGG pathway enrichment of the DEMs.

To provide insights into the molecular mechanism of *L. lancifolium* in response to Se, based on the physiological and growth indices of *L. lancifolium* bulbs, samples from the 0 and 2.0 mmol/L Se treatment groups were subjected to nontargeted metabolome analysis. A total of 969 metabolites were identified. These metabolites were mainly classified into 12 categories: “lipids and lipid-like molecules”, “organic oxygen compounds”, “benzenoids”, “nucleosides, nucleotides, and analogs”, “phenylpropanoids and polyketides”, “organic nitrogen compounds”, “alkaloids and derivatives”, etc. (**Supplementary Figure S2**; **Supplementary Table S5**). Overall, 100 differentially expressed metabolites (DEMs) were screened. The DEMs were divided into two groups according to their expression profiles between the 0 and 2.0 mmol/L Se treatment groups according to PLS-DA and OPLS-DA (**Supplementary Figures S3**, **S4**). These DEMs were categorized into 10 terms, mainly including amino acids (16), steroids and steroid derivatives (9), prenol lipids (8), carbohydrates (4), glycerophospholipids (4) and organic acids and derivatives (4) (**Figure 5A**). Moreover, KEGG pathway enrichment analysis revealed that these DEMs were significantly enriched in glycerophospholipid metabolism, “glycine, serine and threonine metabolism”, “cysteine and methionine metabolism”, and cyanoamino acid metabolism (**Figure 5B**). The upregulated DEM-enriched KEGG pathways were associated mainly with amino acids (L-serine and L-aspartic acid), glycerophospholipids and carbohydrates.

### 3.7 Combined transcriptome and metabolome analysis

**Figure 6:**
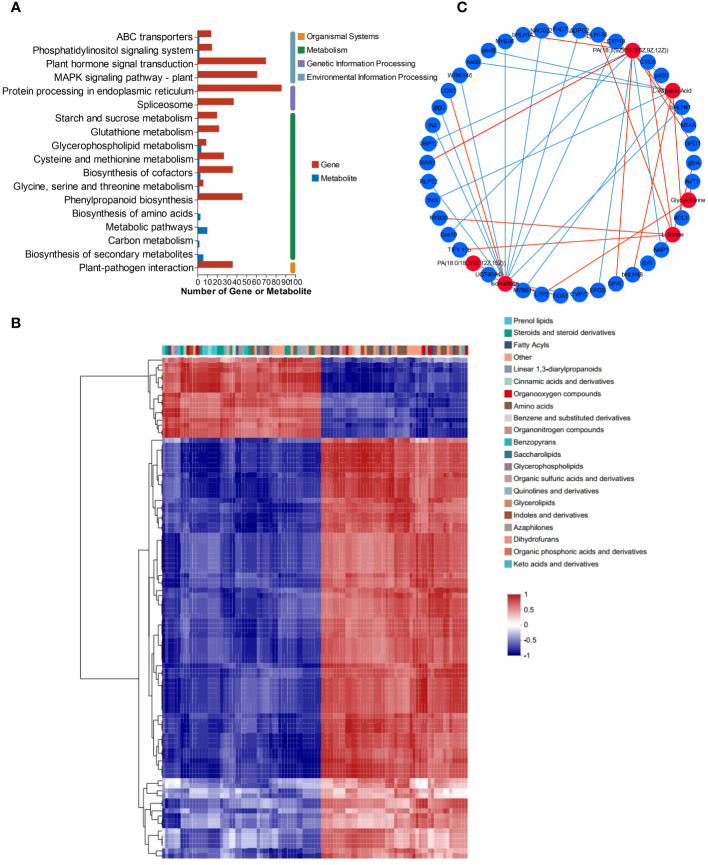
Metabolomic and transcriptomic analysis of *L. lancifolium* under 0 and 2.0 mmol/L Se treatments. **(A)** KEGG pathway enrichment of DEGs and DEMs. **(B)** Clustered heatmap of DEGs and DEMs. **(C)** Interaction network of DEGs and DEMs involved in glycerophospholipid, amino acid and soluble sugar biosynthesis in *L. lancifolium* based on Pearson correlation analysis. The blue circles represent the DEGs, and the red circles represent the DEMs. The blue straight line indicates a negative correlation, and the red straight line indicates a positive correlation.

Based on the transcriptomic and metabolomic data, the KEGG pathways were chosen as the carrier, and an integrative analysis was conducted. The KEGG pathway numbers associated with the transcriptome and metabolome are shown in the Venn diagram (**Supplementary Figure S5**). The cross areas in the circle represent the number of KEGG pathways associated with the differentially accumulated genes and metabolites identified by the two omics methods. All the DEGs and DEMs were enriched according to KEGG pathway enrichment analysis to determine the pathways associated with the DEGs and DEMs. Both the DEMs and DEGs were significantly enriched in 8 KEGG pathways, including glycerophospholipid metabolism (map00564), “glycine, serine and threonine metabolism” (map00260), “cysteine and methionine metabolism” (map00270), “alanine, aspartate and glutamate metabolism” (map00250), and “starch and sucrose metabolism” (map00500) (**Figure 6A**). The DEGs and DEMs in *L. lancifolium* under Se treatment, with a Pearson correlation coefficient (PCC) higher than 0.8, were selected, and a clustered heatmap was drawn. The clustered heatmap showed that the DEMs related to DEGs were classified into several categories, among which amino acids, lipids and carbohydrates were the three largest categories (**Figure 6B**). To study the relationships between DEGs and DEMs in *L. lancifolium* under Se treatment, a PCC *>* 0.90 and a p value *<* 0.05 were used as the threshold values for carrying out co-expression network analysis (**Figure 6C**). These results suggested that Se may be related to complex network regulatory relationships with glycerophospholipid, amino acid, and soluble sugar biosynthesis pathways, which promote the accumulation of these nutrients to improve the growth and nutritional quality of *L. lancifolium*.

**Figure 7:**
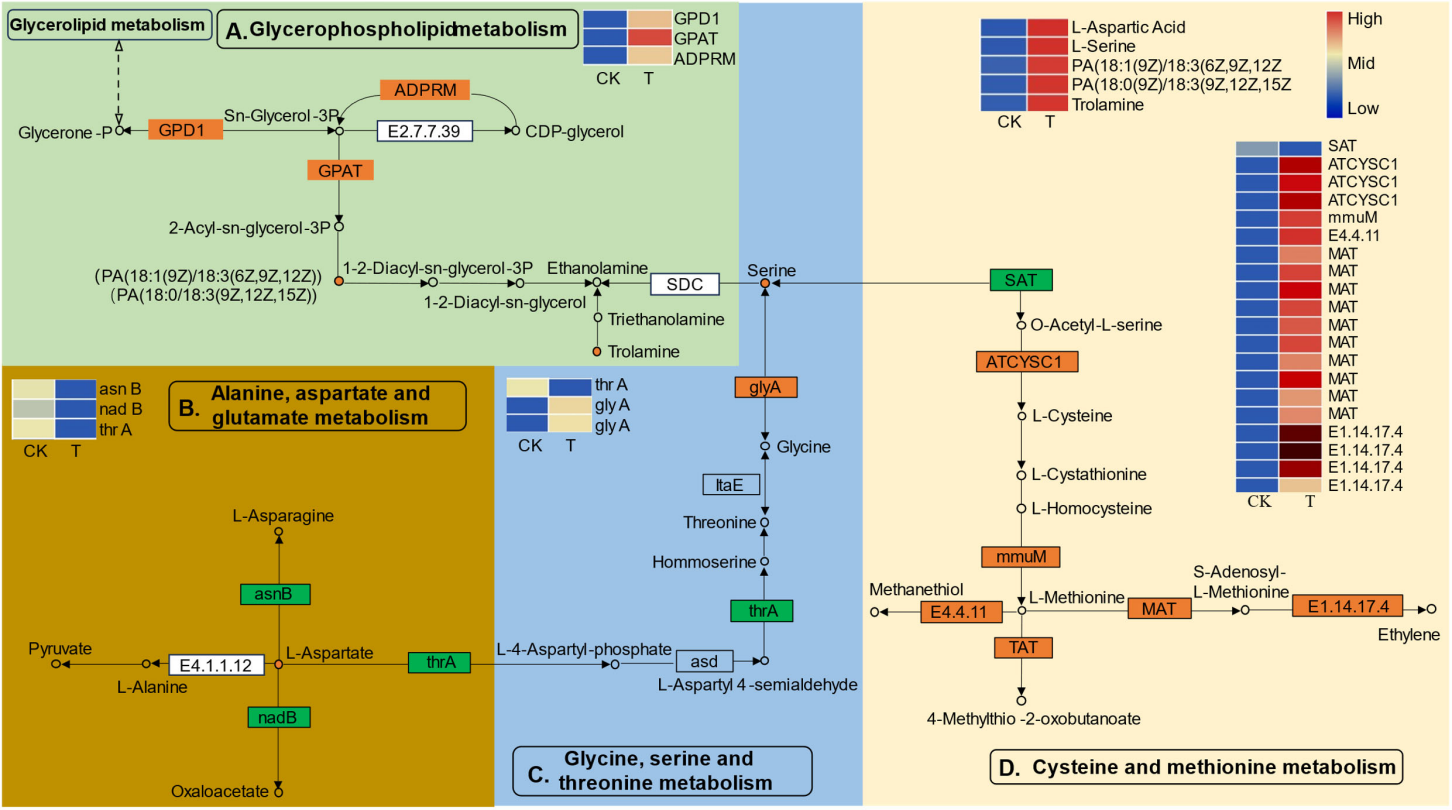
The differentially expressed genes (DEGs) and differentially expressed metabolites (DEMs) involved in the pathways of “glycerophospholipid metabolism **(A)**”, “alanine, aspartate and glutamate metabolism **(B)**”, “glycine, serine and threonine metabolism **(C)**”, and “cysteine and methionine metabolism **(D)**” in *L. lancifolium* responded to Se. The green box represents glycerophospholipid metabolism, the golden box represents alanine, aspartate and glutamate metabolism, the blue box represents glycine, serine and threonine metabolism, and the yellow box represents cysteine and methionine metabolism. The circles denote the metabolites (orange represents upregulation). The rectangles denote the genes (orange represents upregulation; green represents downregulation).

To obtain a deeper understanding of the enriched metabolic pathways, including glycerophospholipid, amino acid, and soluble sugar biosynthesis, based on the combined metabolome and transcriptome analysis, metabolic pathway diagrams were constructed. In the glycerophospholipid metabolism pathway (**Figure 7A**), three genes, namely, *glycerol-3-phosphate dehydrogenase* (*GPD1*), *glycerol-3-phosphate acyltransferase* (*GPAT*), and *manganese-dependent ADP-ribose* (*ADPRM*), were significantly upregulated. Notably, *GPAT* is the first key enzyme involved in the synthesis of glycerides. According to the metabolomics analysis, PA [18:1(9Z)/18:3(6Z,9Z,12Z)] and PA [18:1(9Z)/18:3(6Z,9Z,12Z)], precursor substances of the key substance 1–2-diacyl-sn-glycerol-3P, were found to be highly accumulated during Se treatment. Serine can be decomposed into ethanolamine via a process catalyzed by serine decarboxylase (SDC), and serine is the key substrate for “glycine, serine and threonine metabolism”. Among the genes related to “glycine, serine and threonine metabolism” (**Figure 7C**), the gene encoding aspartokinase/homoserine dehydrogenase (thrA) was significantly downregulated, while two genes encoding serine hydroxy methyltransferase (glyA) were significantly upregulated. glyA catalyzes the reversible simultaneous conversion of L-serine to glycine. As shown by the metabolic analysis, the serine content was strongly increased by the upregulation of glyA. However, the downregulated homoserine dehydrogenase (thrA) was not consistent with the changes in homoserine and threonine levels. In addition, the L-aspartic acid reaction catalyzed by aspartokinase (thrA) links “glycine, serine and threonine metabolism” and “alanine, aspartate and glutamate metabolism” together. L-aspartate is an important precursor for the synthesis of amino acids such as lysine, threonine, isoleucine, methionine, and purine and pyrimidine bases in living organisms. In the present study, genes involved in the biosynthesis and transformation of L-aspartate were identified (**Figure 7B**). Aspartate synthase (asnB) and L-aspartate oxidase (nadB) were significantly downregulated. AsnB catalyzes the biosynthesis of asparagine based on the substrate aspartic acid, and nadB catalyzes the transformation of aspartate to oxaloacetate (Ito et al., 2023). Consistently, the content of the substance L-aspartate strongly increased with decreasing conversion. Serine is also the final reaction product of cysteine and methionine metabolism. Many DEGs related to cysteine and methionine metabolism were identified (**Figure 7D**). Three genes encoding L-3-cyanoalanine synthase/cysteine synthase 2 (ATCYSC1), one gene encoding homocysteine S-methyltransferase 1 (mmuM), ten genes encoding methionine adenosyltransferase (MAT), and two genes encoding nicotianamine aminotransferase 1-like (TAT) were significantly upregulated, while one gene encoding serine acetyltransferase (SAT) was significantly downregulated. Metabolomic analysis revealed that the cysteine and methionine content remained unchanged, which might suggest that the biosynthesis and transformation of cysteine and methionine dynamically changed.

**Figure 8:**
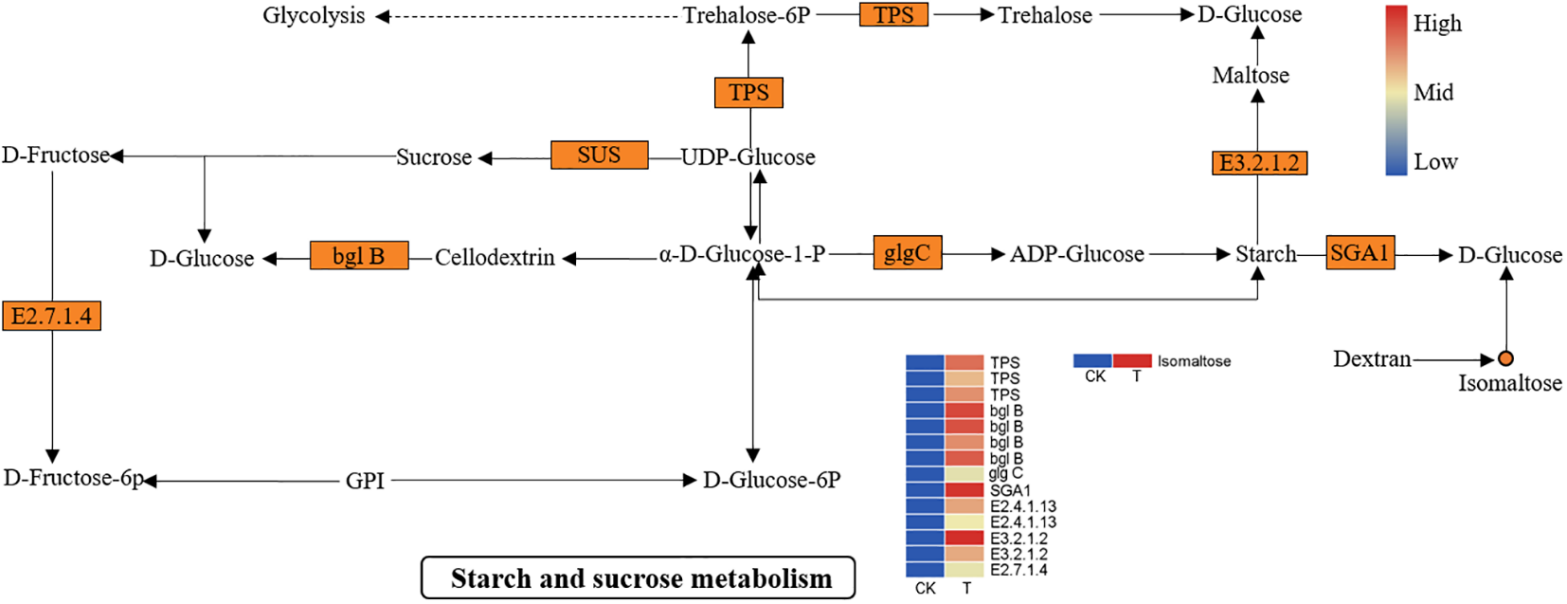
DEGs and DEMs involved in “starch and sucrose metabolism” in *L. lancifolium* in response to Se. The circles denote the metabolites (orange represents upregulation). The rectangles denote the genes (orange represents upregulation; green represents downregulation).

Another important biological process, “starch and sucrose metabolism”, was significantly activated. As shown in **Figure 8**, many DEGs and DEMs were induced in the Se treatment group. The expression of genes such as alpha-trehalose-phosphate synthase (TPS), sucrose synthase (SUS), beta-amylase (E3.2.1.2), fructokinase-1 (E2.7.1.4), beta-glucosidase (bglB), AGPase (glgC), and glucoamylase (SGA1) was significantly upregulated. Metabolic analysis revealed that the isomaltose content was significantly greater in the Se treatment group than in the control group. The upregulation of beta-amylase and glucoamylase plays an important role in the breakdown of amylose to maltose and D-glucose. Similarly, the upregulation of fructokinase-1 and bglB prompts the entry of sucrose into fructose and glucose. The soluble sugar content increased markedly during starch and sucrose metabolism.

### 3.8 Validation of the DEGs by qRT−PCR

**Figure 9:**
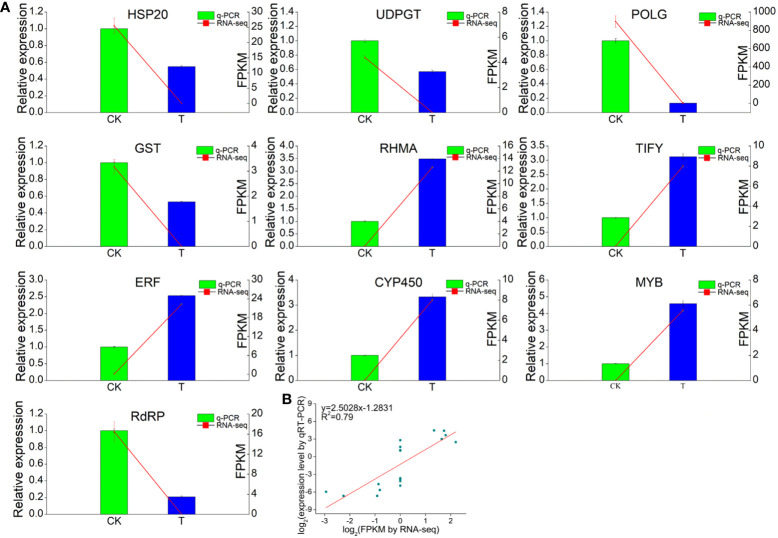
RNA-seq analysis and quantitative RT−qPCR validation of genes differentially expressed between the 0 and 2.0 mmol/L Se treatment groups. **(A)** Correlations between the RNA-seq and RT−qPCR results for the selected individual genes. **(B)** Correlations between the RNA-seq and RT−qPCR results for all the selected genes. *GAPDH* gene was used as the reference gene. Three technical replicates were performed.

The RNA-Seq data were validated by qRT−PCR of ten unigenes related to TFs or Se responses (**Figure 9**). The primers used in this study are shown in **Supplementary Table S6**. The transcript levels of five responsive unigenes (*RdRP*, *HSP*, *UDPGT, POLG* and *GST*) were significantly upregulated in the Se treatment group compared with those in the CK group, while the expression of one gene, *RHMA*, was significantly lower. Three TF (*TIFY*, *ERF* and *MYB*) genes were also evaluated. The expression of all three TFs was markedly upregulated by Se treatment. Additionally, the expression of the cytochrome P450 (CYP450) gene was induced by Se. Furthermore, correlation analysis revealed that the expression levels of these unigenes determined via qRT−PCR were consistent with those determined via RNA−Seq, suggesting that the RNA−seq data were accurate and reliable.

## 4 Discussion

### 4.1 Se regulates the physiological and biochemical traits of *L. lancifolum*

Previous studies have shown that Se application can affect the physiological, molecular and morphological traits of plants (Ekanayake et al., 2015; Schiavon and Pilon-Smits, 2017). In addition, members of Liliaceae family can accumulate large amounts of organic Se more easily than other types of plants (Kavalcová et al., 2014; Sun et al., 2023). Therefore, consuming Se-enriched *L. lancifolium* may be an efficient strategy for addressing Se deficiency problems in selenium-deficient humans. Appropriate concentration of Se can promote plant growth, and the accumulation of soluble sugars, soluble proteins, and other substances, but excessive Se could have opposite effects (Ulhassan et al., 2019; Alves et al., 2020). In the present study, a moderate concentration (2.0 mmol/L) of Se promoted the growth of aboveground parts and bulbs of *L. lancifolium* (**Figures 1**, **2**). Moreover, 2.0 mmol/L Se had a significant effect on bulb biomass and the content of soluble sugars and soluble proteins. However, high Se treatment (≥4.0 mmol/L) inhibited the normal growth and development of *L. lancifolium*, and the leaves became chlorotic and withered (**Figures 1**–**3**). These results indicate that Se has dual effects on *L. lancifolium* plant growth, which is consistent with previous studies. A previous study demonstrated that 0.1–0.4 mmol/L Se promoted cabbage (*Brassica oleracea* var. capitata L.) growth, while 0.4 mmol/L above Se had the opposite effect and the plant showed symptoms of wilting, leaf chlorosis, and senescence (Yang et al., 2022). However, the study on broccoli (*Brassica oleracea* L. var. italica) showed that 0.2–0.4 mmol/L Se significantly decreased the fresh weight of florets (Rao et al., 2021), indicating that different plants have diverse tolerance to selenium.

### 4.2 The influence of Se application on the nutritional quality of *L. lancifolum*

Quality traits such as nutrient availability and flavor are complex properties that are strongly influenced by the composition of soluble sugars, amino acids, organic acids and many other secondary metabolites (Gonçalves et al., 2021; Li et al., 2023). Soluble protein can also be used as an indicator of the nutritional quality and growth status of horticultural crops. For instance, high soluble protein content in vegetables, such as broccoli and Dutch carrot, indicates high nutrient content (Masih et al., 2002; Schiavon et al., 2020). As an edible and medicinal plant, *L. lancifolium* presented increased soluble sugar, soluble protein, and amino acid content in response to the 2.0 mmol/L Se treatment, compared with those of the control. Our results were similar to the findings reported on cabbage and broccoli (Rao et al., 2021; Yang et al., 2022). These findings indicate that appropriate Se application can improve the nutritional quality of *L. lancifolium*. Flavonoids and alkaloids are important secondary metabolites which are beneficial to human health and have advantages in the treatment of diseases (Schiavon et al., 2020). In the present study, 0.5–2.0 mmol/L Se did not affect the flavonoid or alkaloid content of *L. lancifolium*, while ≥4.0 mmol/L Se treatments stimulated the flavonoid and alkaloid accumulation. These results showed that the synthesis of flavonoids and alkaloids in *L. lancifolium* was promoted under excessive Se application, indicating that secondary metabolites are more likely to accumulate under stress conditions, and these findings were similar to those of a previous study on *C. tangshen* (Zhang et al., 2021). In conclusion, an appropriate concentration of Se can improve the quality and yield of *L. lancifolium.*

Many studies have shown that organic Se is far safer and has greater biological potency for humans than inorganic Se (Naiel et al., 2021; Li et al., 2021b; Chen et al., 2022). In the present study, the organic Se content increased with increasing Se concentration, and the organic Se content of the bulbs reached a considerable 3.9 mg/kg (DW). Transformation of organic Se has also been found in Cruciferae plants, such as cabbage and broccoli (Rao et al., 2021; Yang et al., 2022). Interestingly, in our study, the content of organic Se was the same as total Se, indicating that inorganic Se was easily absorbed and totally transformed into organic Se in *L. lancifolium.*

### 4.3 The physiological response of *L. lancifolum* to Se and its potential molecular mechanisms

In this study, combined transcriptomic and metabolic analyses were conducted to elucidate the physiological and molecular mechanisms involved in the response of *L. lancifolium* to Se. The pivotal genes and metabolic pathways involved in glycerophospholipid metabolism, amino acid metabolism, and starch and sucrose metabolism were screened by combined analysis of the transcriptome and metabolome. Glycerophospholipids (GPLs) not only conform to the cell membrane architecture but also provide precursor substances and energy for a wide variety of biological processes, regulating plant growth (Sosa Alderete et al., 2020). Previous studies have shown that glycerol-3-phosphate dehydrogenase (GPD1) is a vital enzyme in lipid synthesis that converts dihydroxyacetone phosphate to sn-glycerol-3-phosphate, and Mn-dependent ADP-ribose/CDP-alcohol diphosphatase (ADPRM) catalyzes the conversion of CDP-glycerol to sn-glycerol-3-phosphate. Then, glycerol-3-phosphate acyltransferase (GPAT) converts sn-glycerol-3-phosphate into 2-acyl-sn-glycerol-3P and 1,2-diacyl-sn-glycerol-3P (Gonzalez-Baro and Coleman, 2017; Koh et al., 2023). In the present study, the *GPD1* and *GPAT* genes were significantly upregulated in the Se-treated group, compared with the control. These genes coordinately promoted the phospholipids biosynthesis of PAs [18:1(9Z)/18:3(6Z,9Z,12Z), 18:1(9Z)/18:3(6Z,9Z,12Z)], which are intermediate substances of 1,2-diacyl-sn-glycerol-3P. Relevant studies have revealed that stimulating denovo phospholipid biosynthesis via gene overexpression can enhance root growth (Angkawijaya et al., 2017; Tan and Nakamura, 2022). Similarly, in this study, the increase of PAs [18:1(9Z)/18:3(6Z,9Z,12Z), 18:1(9Z)/18:3(6Z,9Z,12Z)] might play vital roles in membrane construction and cell biological processes, thus promoting bulb growth in *L. lancifolium*. Taken together, these findings confirmed that *GPD1*, *GPAT* and *ADPRM* are the key genes involved in phospholipid biosynthesis in *L. lancifolium.*

Amino acids are essential compounds involved in the biosynthesis of proteins, the predominant provision of nitrogen (N), and signaling molecules that determine the taste and nutritional value of products (Li et al., 2021a; Tan and Nakamura, 2022). Se can reportedly induce the accumulation of amino acids, sucrose, and storage proteins in plants (Hu et al., 2022; Silva et al., 2023). AsnB is a catalytic enzyme that converts L-aspartate to asparagine, nadB catalyzes the oxidation of L-aspartate to oxaloacetate, and thrA is involved in the conversion of L-aspartate to L-4-aspartate-phosphate (Liu et al., 2022; Ito et al., 2023). The present study showed that the genes *asnB*, *nadB*, and *thrA* were significantly downregulated, thus the degradation of L-aspartate was inhibited, indicating that L-aspartate accumulated markedly at the transcriptional level. Moreover, metabolic analysis confirmed that the abundance of L-aspartate increased in the 2.0 mmol/L Se treatment. The *glyA* gene encodes an *α*-class pyridoxal-5′-phosphate (PLP)-dependent enzyme which catalyzes the conversion of glycine to serine (Hu et al., 2022). In this study, transcriptome analysis revealed that two *glyA* genes were significantly upregulated in 2.0 mmol/L Se treatment, compared with the control, indicating that serine was strongly accumulated in *L. lancifolium* under 2.0 mmol/L Se treatment. These results suggest that Se can upregulate genes like *asnB*, *nadB* and *thrA*, which in turn enhances the biosynthesis of specific amino acids. Similarly, studies on lettuce and tea plants revealed that the content of amino acids, such as glyA, L-aspartate acid and serine, were increased under Se application (Piñero et al., 2022; Xiang et al., 2022). With respect to cysteine and methionine metabolism, transcriptome and metabolism analyses revealed various upregulated DEGs, such as *ATCYSC1*, *mmuM*, *MAT*, *TAT* and *SAT*. However, there was no change in the amino acid content of cysteine and methionine, indicating that the expression of DEGs related to cysteine and methionine metabolism dynamically changed under Se application. These results demonstrated that the *glyA*, *asnB*, *nadB*, *thrA* and *SAT* genes play important roles in Se-induced amino acid biosynthesis, thus improving the nutritional quality of *L. lancifolium*.

Soluble sugars such as sucrose, fructose, glucose and isomaltose are important nutrients which will be increased in plants under appropriate Se treatments (Kaur et al., 2018). Soluble sugar components are involved in starch and sucrose metabolism. Sucrose synthase is an enzyme that can catalyze the synthesis of sucrose from UDP-glucose (Stein and Granot, 2019). In the present study, two *SUS* genes which catalyze sucrose synthesis were significantly upregulated, and sucrose biosynthesis was enhanced, which might gradually promote the growth and development of *L. lancifolium* in response to appropriate Se treatments. Beta-glucosidase (bgl B) is involved in the hydrolysis of cellodextrin into glucose, beta-amylase (E3.2.1.2) converts starch into maltose and glucose, and glucoamylase (SGA1) can catalyze the conversion of starch into glucose (Han et al., 2021; Purwadi et al., 2021; Deflandre et al., 2022). In this study, four *bgl B* genes, two *beta-amylase* genes, and one *SGA1* gene were significantly upregulated, which might promote the glucose accumulation in *L. lancifolium* under Se application. Similarly, the upregulation of genes such as *SUS*, *SPS*, and *bgl B* led to an increase in soluble sugars in apple under appropriate Se application (Liu et al., 2024). Additionally, metabolic analysis revealed that the soluble sugar content of isomaltose was significantly greater in the 2.0 mmol/L Se treatment than that in the control. However, differentially expressed soluble sugars, such as glucose, sucrose and fructose, were not detected in the metabolic analysis, possibly because these substances may be detected with a mass spectrometry peak. Overall, the combined transcriptome and metabolism analysis revealed that genes such as *SUS*, *bgl B*, *BAM*, and *SGA1* played vital roles in the biological process of soluble sugar synthesis, which was consistent with the physiological parameters.

## 5 Conclusion

**Figure 10:**
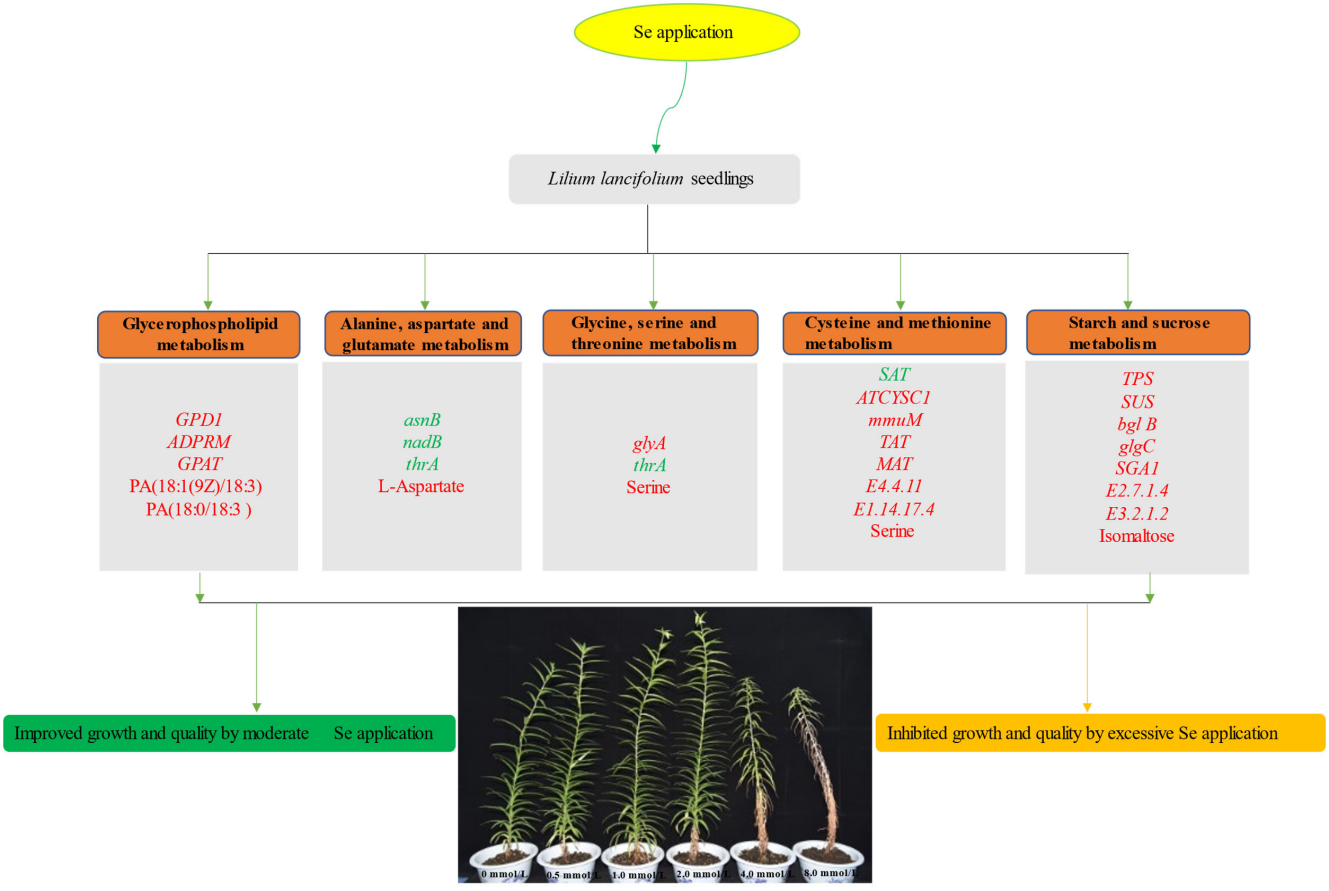
A conclusive model of the response of *L. lancifolium* to different Se treatments. The italic font in the box indicated the genes and non-italic font indicated the metabolites. The genes or metabolites labeled with red were up regulated, while labeled with green were down regulated.

This study explored the effects of different levels of Se on the growth and nutritional quality of *L. lancifolium*. Our results revealed that 2.0 mmol/L Se markedly increased the content of total Se, organic Se, and the main metabolites (phospholipid, amino acids, soluble proteins, soluble sugars), thus comprehensively improving the growth and nutritional quality of *L. lancifolium*. Integrated analysis of the transcriptome and metabolome revealed that Se application positively regulated the metabolic pathways of phospholipid biosynthesis (*GPD1*, *GPAT* and *ADPRM*), amino acid biosynthesis (*glyA*, *asnB*, *nadB*, *thrA* and *SAT*), and starch and sucrose metabolism (*SUS, bgl B, BAM*, and *SGA1*). Appropriate Se application could promote the nutritional quality of *L. lancifolium*, and 2.0 mmol/L Se treatment was the optimal. In summary, this study systematically revealed the relationships among glycerol phospholipid metabolism, amino acid metabolism, and soluble sugar accumulation, and provided new insights into the potential molecular mechanisms underlying Se induced response in *L. lancifolium* (**Figure 10**).

## Data availability statement

The data presented in the study are deposited in the NGDC Genome Sequence Archive (GSA) database, https://ngdc.cncb.ac.cn/, accession number CRA014864.

## Author contributions

WZ: Conceptualization, Supervision, Writing – original draft. XJ: Conceptualization, Data curation, Writing – original draft. DL: Formal analysis, Software, Writing – original draft. HW: Data curation, Validation, Writing – original draft. YY: Methodology, Writing – original draft. JY: Resources, Writing – original draft. HL: Investigation, Writing – original draft. LA: Conceptualization, Writing – original draft. MZ: Supervision, Writing – review & editing.

## Supplementary material

Supplementary Figure S1 The content of organic selenium in *L. lancifolium* bulbs under different concentrations of selenium.

Supplementary Figure S2 The categories of the identified metabolism in *L. lancifolium* bulbs under different concentrations of selenium.

Supplementary Figure S3 The PLS-DA analysis of metabolites in *L. lancifolium* bulbs under different concentrations of selenium.

Supplementary Figure S4 The content of organic selenium in *L. lancifolium* bulbs under different concentrations of selenium.

Supplementary Figure S5 The KEGG pathways number involved in transcriptome and metabolome were shown in the Venn diagram.

Supplementary Table S1 Statistical analysis of the sequencing data.

Supplementary Table S2 The annotation of the unigenes identified in *L. lancifolium*.

Supplementary Table S3 The Go enrichment of unigenes.

Supplementary Table S4 The KEGG enrichment of the DEGs.

Supplementary Table S5 The annotation of all the metabolisms identified in *L. lancifolium*.

Supplementary Table S6 The information of primers used in the qRT-PCR expriment.
