# Supplementary material for: Combined transcriptome and metabolome analyses reveal the effects of selenium on the growth and quality of Lilium lancifolium
Source: Front Plant Sci. 2024 May 17;15:1399152. doi: 10.3389/fpls.2024.1399152 (PMC11140108; doi:10.3389/fpls.2024.1399152)
Supplement: Supplementary Figure S1 — The content of organic selenium in L. lancifolium bulbs under different concentrations of selenium. [file DataSheet_1.doc]

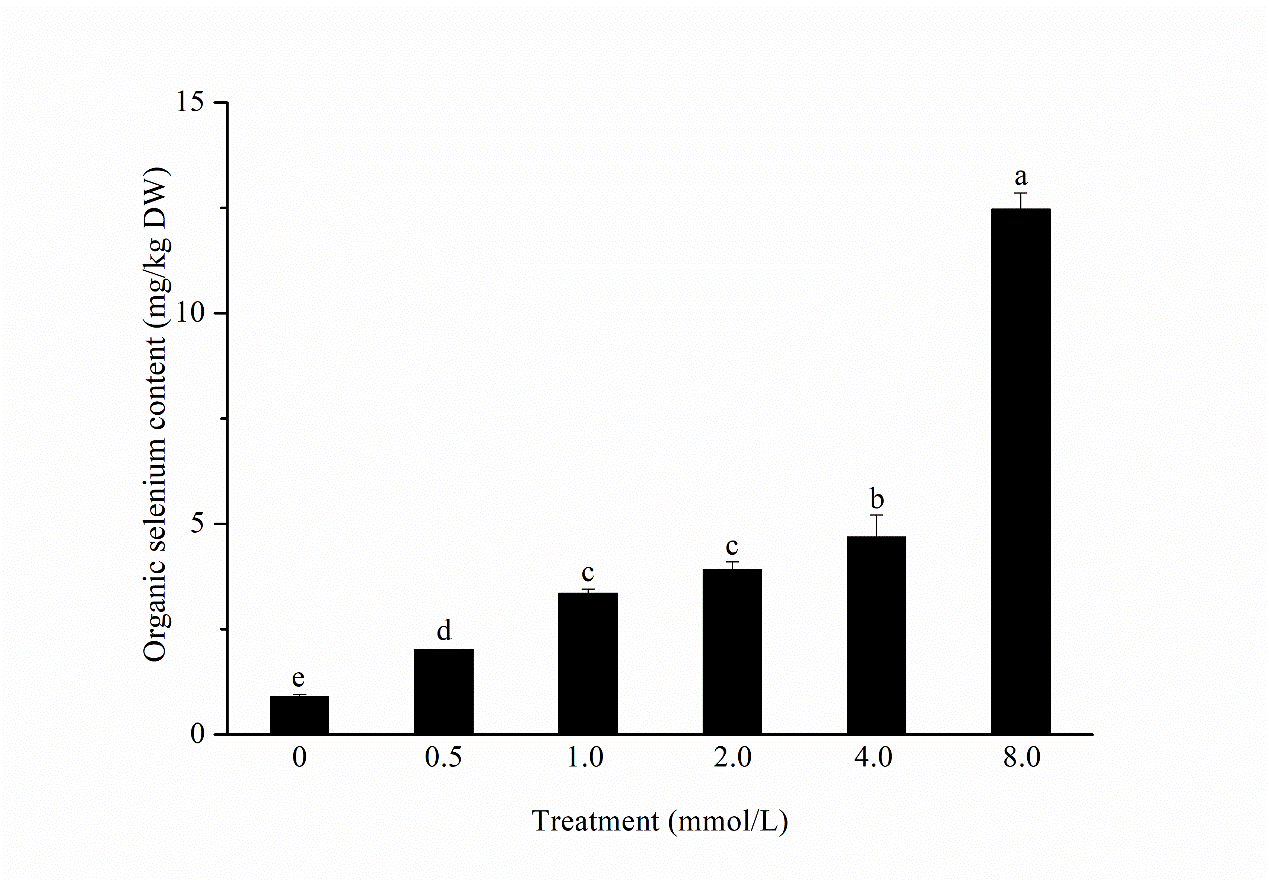


**Fig. S1.** The content of organic selenium in L. lancifolium bulbs under different concentrations of selenium


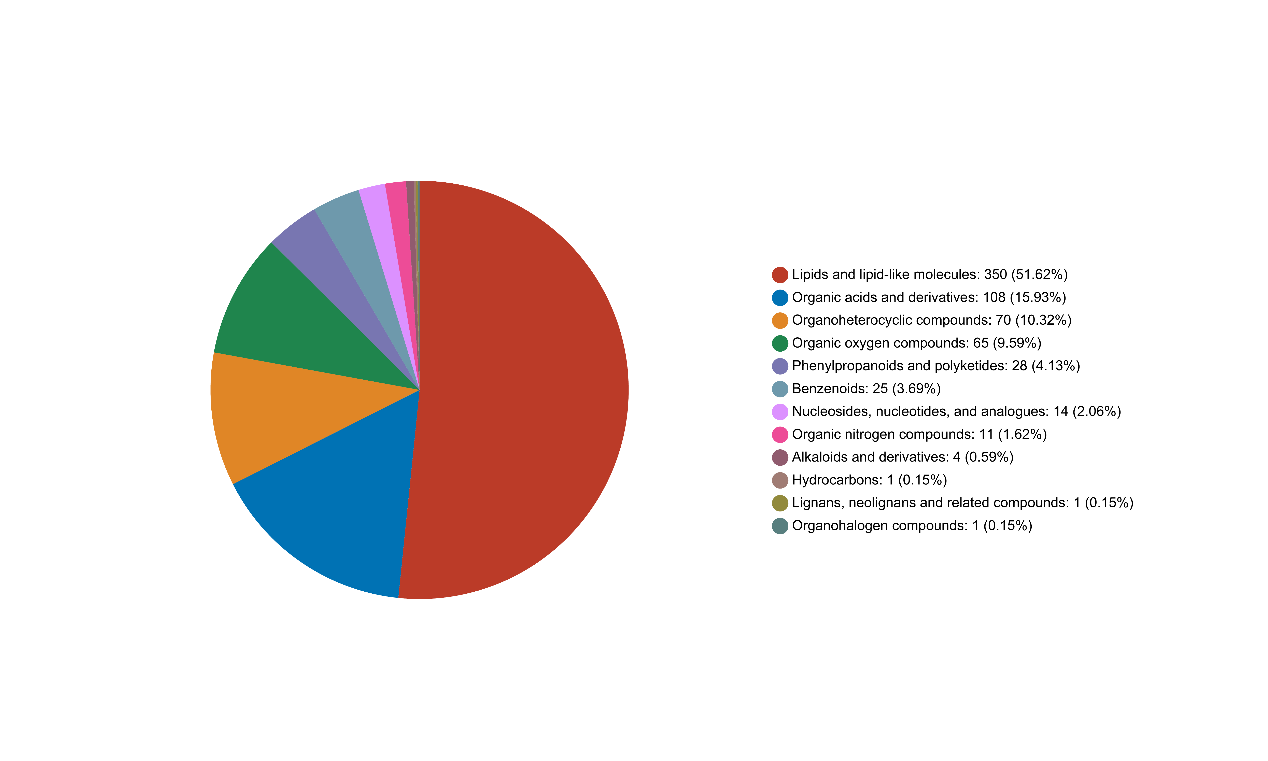


**Fig. S2.** The categories of the identified metabolism in *L. lancifolium* bulbs.


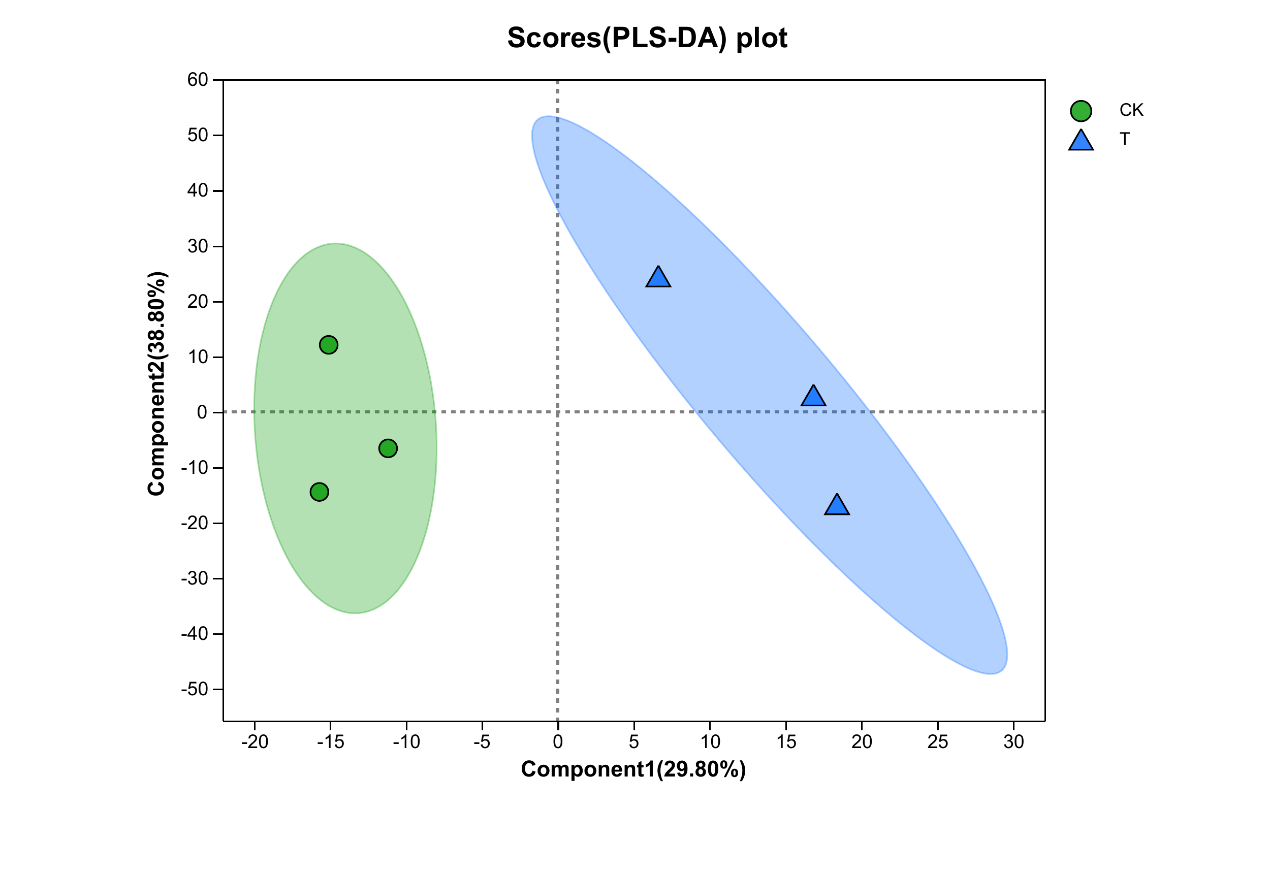


**Fig. S3.** The PLS-DA analysis of metabolites in *L. lancifolium* bulbs in CK and treatment group


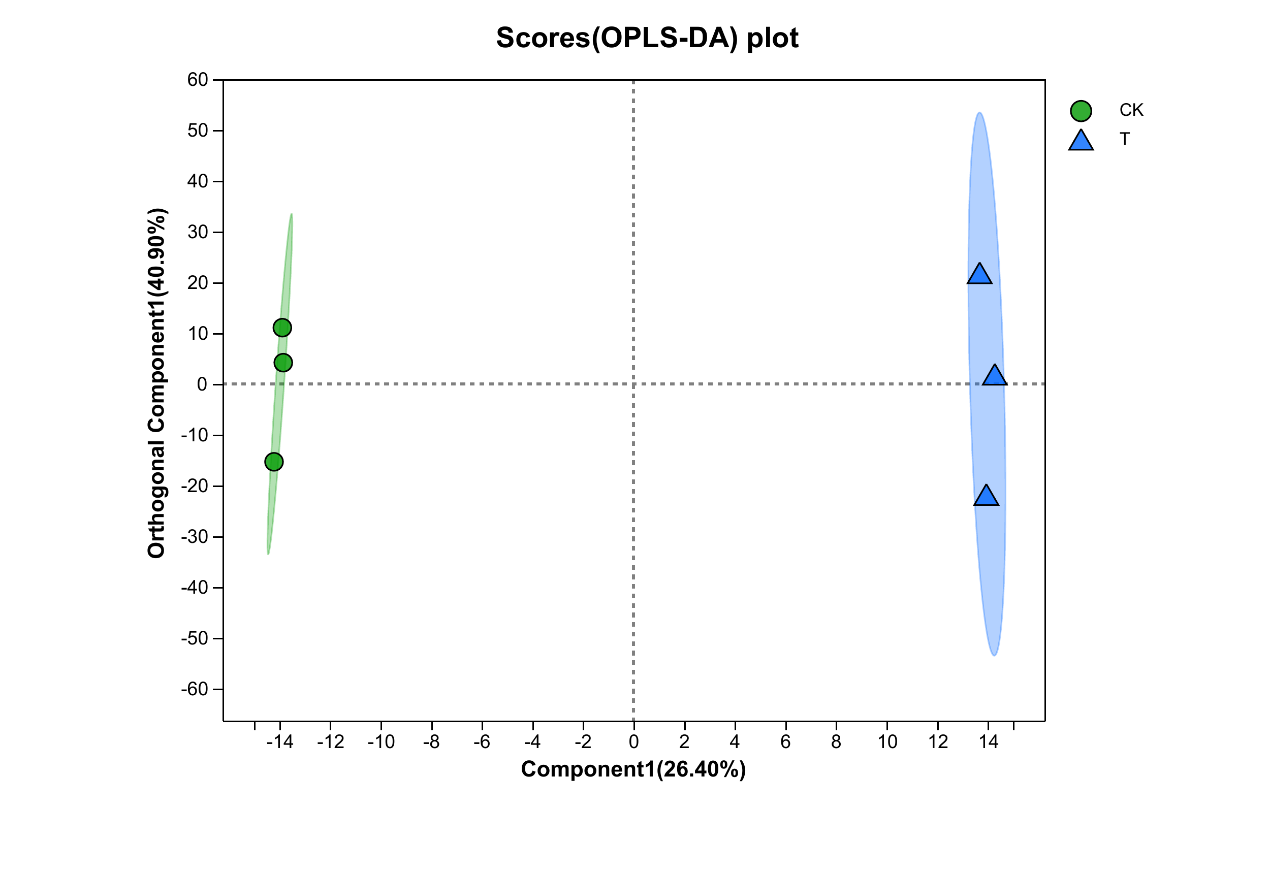


**Fig. S4.** The OPLS-DA analysis of metabolites in *L. lancifolium* bulbs in CK and treatment group


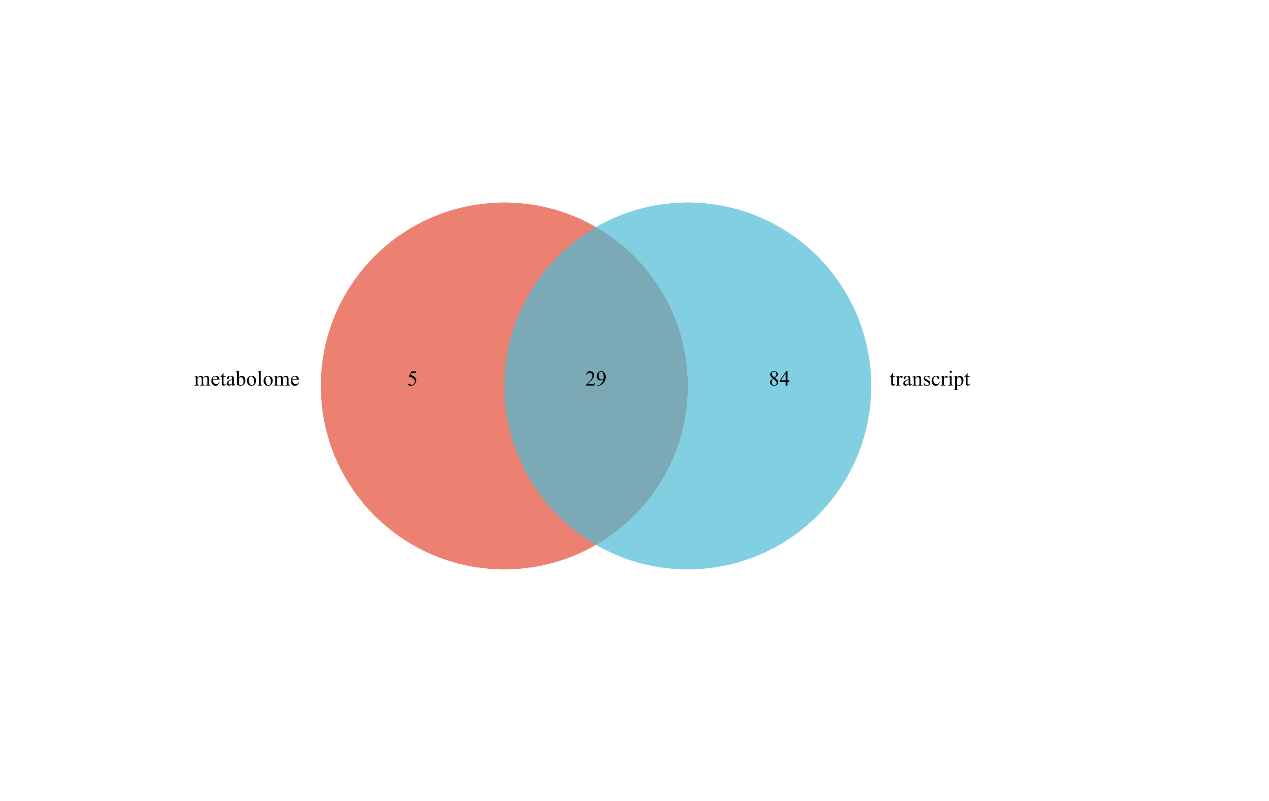


**Fig. S5**. The KEGG pathways number involved in transcriptome and metabolome were shown in the Venn diagram
